# Fibroblast growth factor 21 inhibits atherosclerosis in apoE−/− mice by ameliorating Fas-mediated apoptosis

**DOI:** 10.1186/s12944-018-0846-x

**Published:** 2018-08-29

**Authors:** Xinxin Yan, Zhongshan Gou, Yuan Li, Yu Wang, Jingyan Zhu, Guidong Xu, Qian Zhang

**Affiliations:** 1 Department of Pharmacy, The Affiliated Suzhou Hospital of Nanjing Medical University, 242 Guangji Road, Suzhou, Jiangsu 215008 People’s Republic of China; 2 Center for Medical Ultrasound, The Affiliated Suzhou Hospital of Nanjing Medical University, Suzhou, Jiangsu 215008 People’s Republic of China; 3 Department of Cardiology, The Affiliated Suzhou Hospital of Nanjing Medical University, Suzhou, Jiangsu 215008 People’s Republic of China

**Keywords:** Fibroblast growth factor 21, Fas, Apoptosis, Atherosclerosis

## Abstract

**Background:**

FGF21 is a critical endogenous regulator in energy homeostasis and systemic glucose and lipid metabolism. Despite intensive study of the metabolic functions of FGF21, its important role in heart disease needs further exploration. Apoptosis induced by ox-LDL in vascular endothelial cells is an important step in the progress of atherosclerosis.

**Methods:**

The effects of FGF21 treatment on apoptosis induced by ox-LDL were tested in HUVECs. The role of FGF21 in atherosclerosis was studied by evaluating its function in apolipoprotein E double knockout (apoE−/−) mice.

**Results:**

We found that apoptosis in HUVECs was alleviated by FGF21 treatment. The effects of FGF21 were independent of the ERK1/2 pathway and were mediated through inhibition of the Fas signaling pathway. FGF21 suppressed the development of atherosclerosis, and the administration of FGF21 ameliorated Fas-mediated apoptosis in apoE−/− mice.

**Conclusion:**

FGF21 protects against apoptosis in HUVECs by suppressing the expression of Fas; furthermore, FGF21 alleviated atherosclerosis by ameliorating Fas-mediated apoptosis in apoE−/− mice.

**Electronic supplementary material:**

The online version of this article (10.1186/s12944-018-0846-x) contains supplementary material, which is available to authorized users.

## Background

FGF21 has multiple metabolic functions that occur predominantly in the liver [1] and is a key factor in regulating energy homeostasis and insulin sensitivity [2, 3]. Regulation of lipid and glucose metabolic functions by FGF21 has been reported [4]. FGF21-deficient mice show slightly impaired glucose homeostasis, and when they are raised on a ketogenic diet, they develop hepatosteatosis and obvious impairments in glucose control and ketogenesis [5]. Human recombinant FGF21 has been used to promote glucose incorporation into adipocytes [6], and physiologically, FGF21 has an impact on the metabolic state of starvation, including fatty acid oxidation [7, 8]. Pharmacologically, recombinant FGF21 as a therapeutic intervention was reported to reduce obesity, as well as adiposity, hyperglycemia, and hyperinsulinemia, in both rodents and nonhuman primates [9, 10].

Recently, it was demonstrated that the levels of FGF21 in circulation are promoted in CHD [11] and that FGF21 could attenuate pathological heart remodeling in myocardial infarction [12]. Basic on those studies, FGF21 was suggested to be related to arteriosclerosis, but the effect of FGF21 is still not clear in atherosclerosis, an inflammatory disease that is related to metabolic disorders. Preliminary clinical studies reported that serum FGF21 levels were increased in patients with atherosclerosis and in patients with a high risk of atherosclerosis [13].

Whether the beneficial effects of FGF21 on lipid metabolism are enough to protect against atherosclerosis has not been investigated. Thus, we explored whether FGF21 was involved in the pathogenesis of atherosclerosis in apoE−/− mice. It has been reported that the chronic administration of FGF21 improved HDL cholesterol levels and reduced LDL cholesterol levels in rhesus monkeys and humans with diabetes and obesity [14, 15]. LDL cholesterol particles are transferred to the endoplasmic reticulum, and prolonged ER stress induces apoptosis, which is related to atherosclerotic plaques in apoE-deficient mice [16]. In addition, ox-LDL leads to ER stress-induced death through Fas activation [17]; however, FGF21 in apoE−/− mice reduced ER stress-induced apoptosis [18].

In this study, we investigated the role of FGF21 in suppressing the progression of atherosclerosis, and we tested the hypothesis that FGF21 could inhibit Fas-mediated apoptosis in apoE−/− mice.

## Methods

### Cell culture

Human umbilical vein endothelial cells (HUVECs) were cultured in M199 medium with 15% FBS and 2 mM L-glutamine. HUVECs were treated with 25 μg/ml ox-LDL and 500 ng/ml human recombinant FGF21 (Sigma-Aldrich, MO, USA).

### Animals and ethics statement

ApoE−/− mice on a C57BL6 genetic background and wild-type mice as controls were used. They were fed a high-fat diet with 45% fat, 20% protein, and 35% carbohydrate. They were maintained on a 12 h light/12 h dark cycle at room temperature and had free access to water. Eight-week-old apoE−/− mice were fed a high-fat diet for 12 weeks, and some were intraperitoneally injected with recombinant mouse FGF21 (1.0 mg/kg) or saline daily for 8 weeks.

This study was approved by The Affiliated Suzhou Hospital of Nanjing Medical University Animal Care and Use Committee and conducted in accordance with the “Guide for the Care and Use of Laboratory Animals” set forth by the Jiangsu Province Government.

### Ox-LDL preparation

Ox-LDL, which is highly oxidized LDL, was purchased from Shanghai Jingke Chemical Technology Co., LTD, Shanghai, China. According to the manufacturer, this ox-LDL is extensively oxidized with Cu_2_SO_4_ (oxidant) in PBS at 37 °C and is used to induce cell apoptosis/death and injury.

### FGF21 measurement

Serum levels of FGF21 were measured with a commercially available enzyme-linked immunosorbent assay according to the manufacturer’s instructions (BioVendor, Modrice, Czech Republic).

### siRNA transfection

HUVECs were transfected by Lipofectamine 2000 transfection reagent (Invitrogen, CA) with specific siRNA oligomers against Erk1/2 (100 nM). After transfection for 50 h, the cells were pretreated with FGF21 for 6 h and then treated with ox-LDL (25 μg/ml) for 36 h. Western blotting was used to validate Erk1/2 silencing.

### Western blot

HUVECs were lysed in RIPA buffer, and the lysates were subjected to SDS-PAGE and then transferred to a PVDF membrane. Anti-Fas (rabbit polyclonal IgG), anti-FADD (rabbit polyclonal IgG), anti-GAPDH (mouse monoclonal IgG1 FL rabbit GAPDH) and anti-β-actin (mouse monoclonal IgG) antibodies were used at dilutions of 1:1000, 1:1000, 1:1500 and 1:2000, respectively. A horseradish peroxidase-conjugated secondary antibody was used, and the membrane was detected.

### Apoptosis assay

For the flow cytometry analyses, HUVEC suspensions were stained with annexin V-FITC and propidium iodide (Annexin V Apoptosis Detection Kit FITC, eBioscience). Data were collected on a FACScan cytometer (Aria, BD, USA) and analyzed using CellQuest software.

HUVEC apoptosis was also determined by a Caspase-3/FLICE Colorimetric Protease Assay (Invitrogen). HUVECs were added to a protein lysis buffer, and caspase-3 activity was assessed by measuring the released pNA at OD 405 nm using a spectrophotometric plate reader (Bio-Rad).

### Immunohistochemistry (IHC) staining

Tissue samples were fixed with 4% paraformaldehyde for more than 12 h, embedded in OCT reagent paraffin, and then cut into 15 μm sections for hematoxylin and eosin (HE) staining. For immunostaining of activated FGF21 in OCT samples, a rabbit antibody against FGF21 (Santa Cruz, CA) at a dilution of 1:50 was used.

### Statistics

All experiments were repeated at least three times. Data were presented as the mean ± SD. Differences among groups of 3 or more were assessed with one-way ANOVA and post hoc testing. Percentage and Proportions are analyzed by non-parametric tests. *P* values less than 0.05 were considered to indicate statistical significance.

## Results

### FGF21 inhibits HUVEC apoptosis induced by ox-LDL

**Fig. 1:**
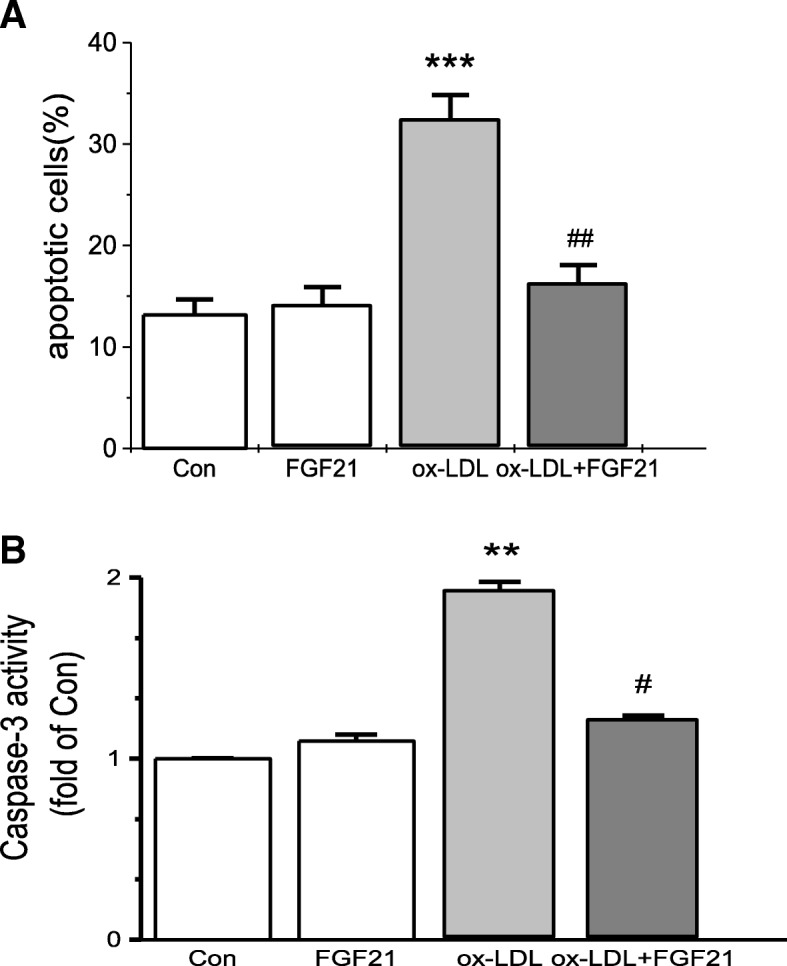
FGF21 protected against ox-LDL-induced HUVEC apoptosis. After pretreatment with 500 ng/ml FGF21 for 6 h, HUVECs were incubated with 25 μg/ml ox-LDL for 36 h. **a** Apoptosis was detected by flow cytometry in HUVECs incubated with ox-LDL with or without FGF21. **b** Apoptosis was detected by using a caspase-3 activity kit for HUVECs incubated with ox-LDL with or without FGF21. ***p < 0.001, **p < 0.01 vs. Con; ##p < 0.01, #*p* < 0.05 vs. ox-LDL

Apoptosis is important in the process of atherosclerosis, and thus, whether FGF21 inhibits HUVEC apoptosis was explored. Apoptosis in HUVECs treated with ox-LDL increased significantly as demonstrated by the flow cytometric analysis results (Fig. 1a). Consistent with the flow cytometric data, apoptosis in HUVECs increased significantly as measured by caspase-3 activity (Fig. 1b). When HUVECs were incubated with FGF21, apoptosis induced by ox-LDL intake in these cells was reduced significantly (Fig. 1a, b). These results suggest that FGF21 inhibits HUVEC apoptosis.

### FGF21 inhibits apoptosis independent of the ERK pathway

**Fig. 2:**
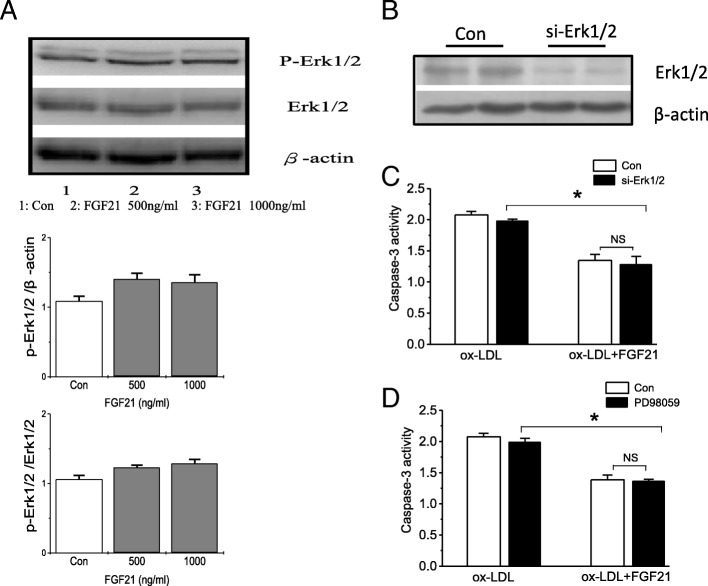
The effect of FGF21 in HUVECs is independent on the ERK signaling pathway. **a** Phosphorylated ERK1/2 levels were measured and quantified by western blotting HUVECs treated with FGF21 (500 ng/ml or 1000 ng/ml) for 36 h. **b** The efficiency of ERK1/2 was detected in HUVECs transfected with siRNA knock down of ERK1/2. **c** The caspase-3 activity was measured in HUVECs treated with ox-LDL with or without FGF21 and with siRNA knockdown of ERK1/2 (**d**) or by p-ERK1/2 inhibitor. *p < 0.05 vs. ox-LDL, N.S: no significance

The ERK signaling pathway has an important effect on FGF21 function [19, 20]. To explore whether HUVEC apoptosis affected by FGF21 was dependent on ERK signaling, HUVECs were incubated with different concentrations of FGF21 (500 ng/ml and 1000 ng/ml). The protein expression of p-ERK1/2, ERK1/2 and p-ERK/ERK was not increased significantly in the FGF21-treated groups, which indicated that FGF21 had no obvious impact on the ERK signaling pathway in HUVECs (Fig. 2a). To further investigate whether the inhibitory role of FGF21 in apoptosis of HUVECs was dependent on the ERK pathway, ERK1/2 was selectively knocked down in HUVECs by siRNAs, and a p-ERK1/2 inhibitor (PD98059) was used to inhibit protein expression. The western blot results demonstrated that ERK1/2 expression was reduced significantly in si-ERK1/2-transfected HUVECs (Fig. 2b). Compared to the ox-LDL-incubated cells, FGF21 reversed apoptosis in Con and si-ERK1/2-transfected cells (Fig. 2c). However, the knockdown of ERK1/2 did not influence apoptosis in HUVECs. Furthermore, to analyze whether the ERK1/2 signaling pathway is involved or not, HUVECs were incubated with PD98059. The ERK1/2 signaling pathway was hindered because of the reduced p-ERK1/2 expression induced by the addition of PD98059 (Additional file 1: Figure S1). However, apoptosis in the cells remained unchanged compared to that in the Con group whether incubated with FGF21 or not (Fig. 2d). These results reveal that the effect of FGF21 in HUVECs is not dependent on the ERK signaling pathway.

### FGF21 inhibits ox-LDL-induced Fas expression

**Fig. 3:**
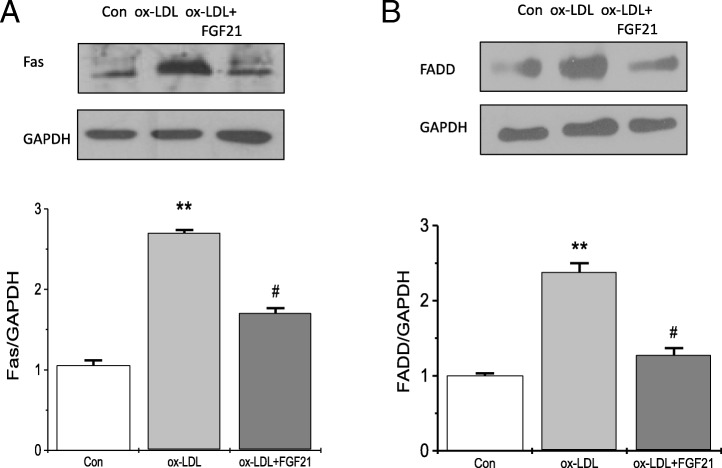
The effect of FGF21 on apoptosis is dependent on the Fas signaling pathway in HUVECs. After pretreatment with 500 ng/ml FGF21 for 6 h, HUVECs were incubated with 25 μg/ml ox-LDL for 36 h. **a** Fas protein levels in HUVECs incubated with ox-LDL with or without FGF21 were measured by western blotting. **b** FADD protein levels in HUVECs incubated with ox-LDL with or without FGF21 were measured by western blotting. **p < 0.01 vs. Con, #p < 0.05 vs. ox-LDL

The apoptosis of HUVECs has been thought to activate extrinsic apoptotic pathways, so we examined the death receptor Fas and the specific adaptor molecule FADD, which are considered as key players in the process of apoptosis. Interestingly, after FGF21 treatment, the protein levels of Fas and FADD induced by ox-LDL in HUVECs were decreased dramatically. As shown by our data, FGF21 dramatically suppressed ox-LDL-induced Fas and FADD expression (Fig. 3).

### FGF21 inhibits atherosclerosis by ameliorating Fas-mediated apoptosis in apoE−/− mice

**Fig. 4:**
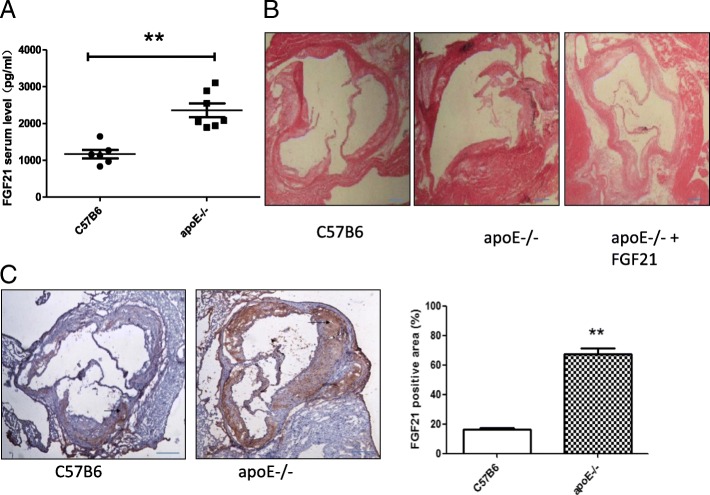
FGF21 attenuated the progress of atherosclerosis. **a** The level of serum FGF21 increased in the apoE−/− mice compared with C57BL6 mice as measured by ELISA. **p < 0.01 vs. C57BL6. **b** The expression of FGF21 in the aortic root region of the heart increased in apoE−/− mice compared with that in C57BL6 mice assessed by IHC analyses (*n* = 6). **p < 0.01 vs. C57BL6. **c** FGF21 reversed the smooth muscle arrangement disorder and thickening and the plaque formation in atherosclerosis of apoE−/− mouse hearts detected by HE staining (n = 6)

Dramatically increased FGF21 levels have been reported in atherosclerosis patients in some clinical studies [11, 13]. Consistent with these data, we found that circulating levels of FGF21 as well as protein expression levels in the heart were correspondingly upregulated in apoE−/− mice with the progression of atherosclerosis (Fig. 4a, c). To further study the effect of FGF21 on atherosclerosis, apoE−/− mice fed the high-fat diet were treated with FGF21, and atherosclerosis in these mice was found to be significantly ameliorated by FGF21 treatment as shown by HE staining of the aorta (Fig. 4b). These results suggest that FGF21 could suppress the development of atherosclerosis in apoE−/− mice.

**Fig. 5:**
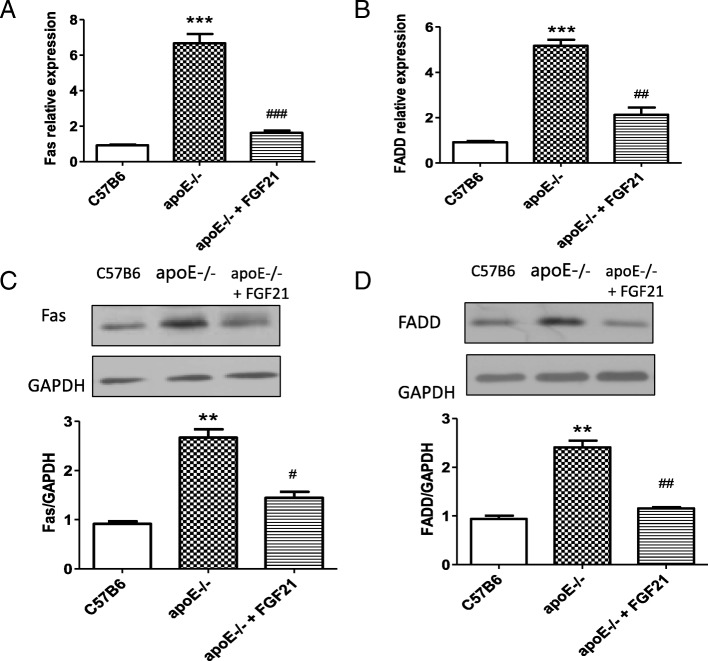
The effect of FGF21 on apoptosis is dependent on the Fas signaling pathway in apoE−/− mice. **a**, **b** Fas and FADD mRNA relative expression was measured and quantified in the aortic root region of hearts from apoE−/− mice treated with or without FGF21. **c, d** Fas and FADD protein levels in the aortic root region of hearts from apoE−/− mice treated with or without FGF21 were measured and quantified by western blotting. ***p < 0.001, **p < 0.01 vs. C57BL6 mice; ###p < 0.001, #p < 0.05 vs. apoE−/− mice

To further study whether FGF21 inhibits atherosclerosis via ameliorating Fas-mediated apoptosis in apoE−/− mice, we measured the mRNA and protein expression of Fas and FADD in the apoE−/− mice and apoE−/− mice treated with FGF21. The data showed that the mRNA and protein expression of Fas in FGF21-treated apoE−/− mice was dramatically decreased compared with that of the control group (Fig. 5a and c). Similarly, the mRNA and protein expression levels of FADD were suppressed in apoE−/− mice treated with FGF21 (Fig. 5b and d). Taken together, these results show that FGF21 alleviated the progression of atherosclerosis by suppressing Fas-mediated apoptosis in apoE−/− mice.

## Discussion

FGF21, a hormone-like factor, has been found to be a metabolic regulator and a new direction for the treatment of diabetes and cardiovascular disease [21, 22]. Despite intensive study of the metabolic functions of FGF21, its important role in atherosclerosis needs further exploration.

Our results show that FGF21 could protect HUVECs against ox-LDL-induced apoptosis and provide evidence that FGF21 hinders the exacerbation of atherosclerosis in apoE−/− mice, revealing that FGF21 is a metabolic disorder regulator for cardiovascular disease. Consequently, elevated circulating FGF21 levels may represent a feedback defense mechanism of the body in response to vascular damage in rodents with atherosclerosis. In support of this idea, several studies reported that elevated serum FGF21 levels have been associated with the presence of CHD and carotid artery plaques in patients [11, 15, 23]. Consistent with these studies, we showed that elevated FGF21 acts as an effective compensatory mechanism to prevent endotoxin-induced apoptosis. Moreover, another study reported that when cardiac microvascular endothelial cells were treated with ox-LDL, the level of FGF21 was elevated, and cell apoptosis was decreased [24]. The research proposes that in the cardiovascular system, FGF21 acts as an endogenous protective factor to improve endothelial function during the early stages of atherosclerosis, which is consistent with our results. Thus, these studies demonstrate that the influence of FGF21 on endothelial cells is indirect, mediated partly through the Fas signaling pathway.

FGF21 is shown to be protective against obesity and atherosclerosis. We used an in vitro and in vivo approach to study the role of FGF21 in the progression of atherosclerosis-associated apoptosis. Major findings of this work include the observation that FGF21 treatment reduces ox-LDL-induced apoptosis, and this process is not dependent on ERK. Most interestingly, we demonstrated that FGF21 treatment reduced the expression of Fas and FADD in both ox-LDL-treated HUVECs and apoE−/− mouse aortic tissue. We speculate that FGF21 prevents atherosclerosis through a mechanism that involves reducing Fas/FADD-mediated apoptosis in endothelial cells. This work is a nice extension of the study recently published by Lin et al., who reported that FGF21 protects against atherosclerosis by modulating adiponectin and SREBP2 levels [25]. Our work provides new insight into an additional mechanism by which FGF21 can prevent atherosclerosis. Furthermore, Xi Wu et al. [18] have reported a cardioprotective effect of FGF21, which is consistent with our results. In their study, the protection against atherosclerosis by FGF21 might be in part due to its inhibitory effects on ER stress-mediated apoptosis, and this mechanism of FGF21 is different from that found in our results. We found that the protective effect of FGF21 against atherosclerosis might be due to its inhibitory effects on Fas/FADD-mediated apoptosis. Furthermore, Xi Wu et al. used apoE−/− mice as their models, and we used not only apoE−/− mice but also an ox-LDL-induced HUVEC apoptosis model to study the signaling pathway.

Dyslipidemia, particularly elevated and oxidized LDL cholesterol concentrations, is an important contributor to the formation of atherosclerotic plaques [26]. Many statins, clinical cholesterol-lowering drugs, have been applied to decrease the risk of hypercholesterolemia, atherosclerosis and CHD. The elevated FGF21 expression in apoE−/− mice suggests that endogenous FGF21 is a physiological inhibitor of cholesterol. FGF21 was demonstrated to counteract the injury induced by elevated and oxidized LDL cholesterol concentrations in rodents [9] and patients with type 2 diabetes [27]. Consistent with these pharmacological results, we found that administration of FGF21 to apoE−/− mice inhibits the further aggravation of ox-LDL-induced Fas signaling apoptosis. In addition, FGF21 also increases HDL, which is known to be an anti-atherosclerotic molecule [28, 29]. Therefore, our current study provides the possibility that FGF21 may be effective for the prevention and treatment of atherosclerosis.

There are several limitations. Although the data demonstrated the function of FGF21 via the suppression of Fas expression in the heart, the signaling pathway that links apoptosis with its other receptors needs further investigation. Our observations are solely based on HUVECs and apoE−/− mice treated with FGF21 and remain to be confirmed in FGF21-deficient animals and clinical studies.

## Conclusions

In summary, we described the effect of FGF21 in suppressing apoptosis induced by ox-LDL in HUVECs and the progression of atherosclerosis. In addition, apoptosis in HUVECs was induced by FGF21 incubation, and while the influence of FGF21 was independent of ERK1/2 signaling, it was found to occur through the inhibition of Fas expression in apoE−/− mice. These results provide important evidence for the role of FGF21 in the development of arteriosclerosis and provide a potential target for atherosclerosis treatment and prevention.

## Additional file

Additional file 1: **Figure S1.** Phosphorylated ERK1/2 and ERK1/2 levels were measured and quantified by western blotting in HUVECs treated with a p-ERK1/2 inhibitor (PD98059). **p < 0.01 vs. Con, N.S: no significance. (DOCX 172 kb)

## Abbreviations

apoE−/− mice: Apolipoprotein E double knockout mice
CHD: Coronary heart disease
ERK1/2: Extracellular regulated protein kinases 1/2
FADD: Fas-associating protein with a novel death domain
FAS: Factor-associated suicide
FBS: Fetal bovine serum
FGF21: Fibroblast growth factor 21
HDL: High-density lipoprotein
HE staining: Hematoxylin and eosin staining
HUVECs: Human umbilical vein endothelial cells
LDL: Low-density lipoprotein
ox-LDL: Oxidized low-density lipoprotein
p-ERK1/2: Phosphorylated extracellular regulated protein kinases 1/2
