# Supplementary material for: Fibroblast growth factor 21 inhibits atherosclerosis in apoE−/− mice by ameliorating Fas-mediated apoptosis
Source: Lipids Health Dis. 2018 Aug 29;17:203. doi: 10.1186/s12944-018-0846-x (PMC6114502; doi:10.1186/s12944-018-0846-x)
Supplement: Supplementary file 1 [file 12944_2018_846_MOESM1_ESM.docx]

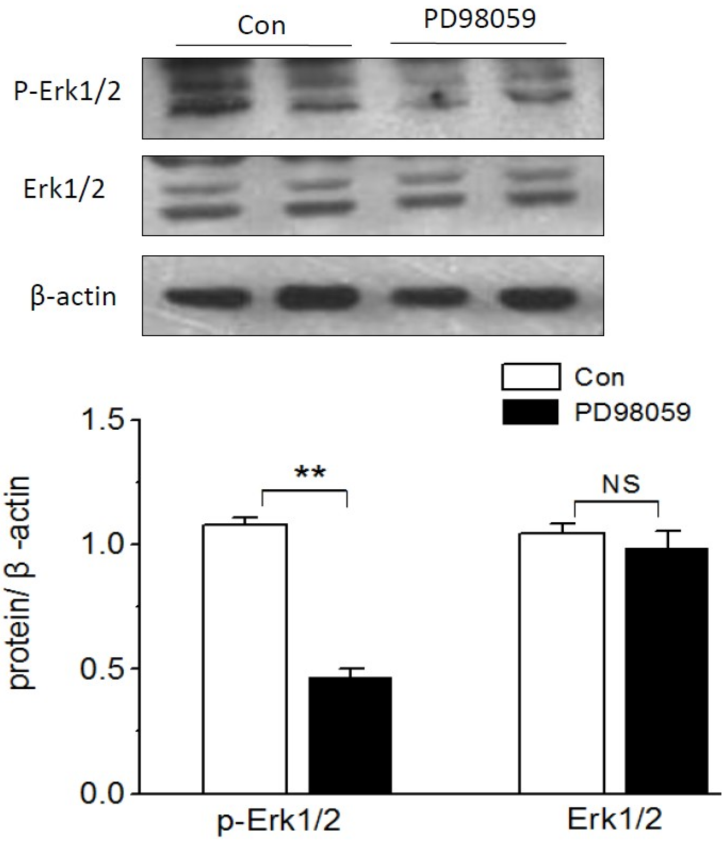

Con
PD98059
P-Erk1/2
Erk1/2
β-actin
Con
PD98059
protein/ β -actin
1.5
1.0
0.5
0.0
**
NS
p-Erk1/2
Erk1/2

Figure S1
